# Association Between Cervical Cancer Screening Guidelines and Preterm Delivery Among Females Aged 18 to 24 Years

**DOI:** 10.1001/jamahealthforum.2023.1974

**Published:** 2023-07-21

**Authors:** Rebecca A. Bromley-Dulfano, Maya Rossin-Slater, M. Kate Bundorf

**Affiliations:** 1 Stanford University School of Medicine, Stanford, California; 2 Now with Department of Health Care Policy, Graduate School of Arts and Sciences, Harvard University Medical School, Cambridge, Massachusetts; 3 Department of Health Policy, Stanford University School of Medicine, Stanford, California; 4 Sanford School of Public Policy, Duke University, Durham, North Carolina

## Abstract

**Importance:**

Cervical cancer screening is associated with reduced cervical cancer mortality; however, clinical trials have also shown an association between excisional procedures for cervical neoplasia and an increased risk of preterm delivery (PTD). National screening guidelines must weigh adverse effects on birth outcomes against benefits of cancer prevention.

**Objective:**

To ascertain the population-level association between the number of guideline-recommended cervical cancer screenings and downstream PTD risk among females aged 18 to 24 years.

**Design, Setting, and Participants:**

This cross-sectional study used a difference-in-differences approach based on variation in the recommended number of cervical cancer screenings (over time and across individuals giving birth at different ages) to estimate the association between the cumulative recommended number of screenings (by the time of childbirth) and PTD risk. National Vital Statistics System data from females aged 18 to 24 years who had a singleton, nulliparous birth in the US between 1996 and 2018 were used. Regression models were adjusted for maternal educational level, race and ethnicity, comorbidities, marital status, and prenatal care visits. Data were analyzed between June 2020 and March 2023.

**Exposure:**

A constructed variable capturing the cumulative number of guideline-recommended cervical cancer screenings for an individual based on their age and year of childbirth.

**Main Outcomes and Measures:**

Binary indicators for PTD and very preterm delivery (VPTD), defined as delivery before 37 and 34 weeks’ gestational age, respectively, and gestational age was measured in weeks from the first day of the last menstrual period.

**Results:**

Among 11 333 151 females aged 18 to 24 years who gave birth between 1996 and 2018, 2 069 713 were Black (18.3%), 2 601 225 were Hispanic (23.0%), 6 068 498 were White (53.5%) individuals, and 593 715 (5.2%) were individuals of other race or ethnicity (Alaska Native; American Indian; Asian; Pacific Islander; multiracial; or unknown or missing race or ethnicity). Mean (SD) age was 20.9 (1.9) years, and 766 001 individuals (6.8%) had hypertension or diabetes. The mean (SD) number of guideline-recommended screenings by time of childbirth was 2.4 (2.2). Overall, PTD and VPTD occurred in 1 140 490 individuals (10.1%) and 333 040 (2.9%) of births, respectively. One additional recommended screening was associated with a 0.073 (95% CI, 0.026-0.120) percentage-point increase in PTD risk but no statistically significant change in VPTD risk. Females with hypertension or diabetes had a higher increase in PTD risk than those without these comorbidities (0.26 [95% CI, 0.11-0.4] vs 0.06 [95% CI, 0.01-0.10] percentage points; Wald test *P* < .001).

**Conclusions and Relevance:**

Findings of this cross-sectional study suggest that additional recommended cervical cancer screenings before birth were associated with an increased risk of PTD. These results can be used in future simulation models integrating oncological trade-offs to help ascertain optimal screening strategies.

## Key Points

**Question:**

What is the population-level association between the number of guideline-recommended cervical cancer screenings and the downstream risk of preterm delivery (PTD) among females aged 18 to 24 years?

**Findings:**

Using US data from 11 333 151 singleton, nulliparous births to females aged 18 to 24 between 1996 and 2018 and difference-in-differences methodology, this cross-sectional study found that an increase in the recommended number of cervical cancer screenings was associated with an increase in PTD risk. Females with hypertension or diabetes had an increased risk of PTD compared with females without these conditions.

**Meaning:**

Cervical cancer screening guidelines should consider the downstream implications for PTD risk when weighing the population-level costs of screenings against the benefits of reduced cervical cancer mortality.

## Teaser

This cross-sectional study examines the association between the number of guideline-recommended screenings for cervical cancer and the risk of preterm delivery in females aged 18 to 24 years.

## Introduction

Early cervical cancer screenings and cervical intraepithelial neoplasia (CIN) treatment are associated with reduced cancer rates and mortality.^1,2^ However, the excisional procedures that patients with abnormal cervical cancer screening results undergo are associated with an increase in the subsequent risk of preterm delivery (PTD) and other adverse perinatal outcomes in clinical trials.^3,4,5^ Thus, US cervical cancer screening guidelines must weigh the oncological benefits for individuals with a cervix (IWCs) against the potential adverse neonatal outcomes.

Cervical cancer prevention begins with a Papanicolaou test. If indicated, patients then undergo a colposcopy, perhaps a biopsy, and, if a high-grade lesion (CIN grade 3 or higher) is present, a cervical excision procedure.^6,7^ Because clinicians have limited ability to predict whether low- to mid-grade lesions will remain benign or become cancerous, many practitioners excise CIN grade 2 or higher lesions precautionarily.^8^ However, in IWCs aged 18 to 24 years, previous studies estimate between 60% and 90% of these lesions (even high-risk subtypes) regress within 2 years, rendering some excisional treatments unnecessary.^9,10,11,12^ Studies suggest that pregnant IWCs with a history of CIN are already at increased risk for PTD, and excisional treatments may further increase this risk.^4,13,14,15,16,17^ Increasing excision depth and proximity to treatment are associated with worse outcomes. Additional risk factors associated with PTD include low socioeconomic status, maternal age, experiencing racism (particularly anti-Black racism),^18,19^ stress, depression, tobacco use, and assisted reproductive technology use.^20,21^

The optimal screening strategy for cervical cancer must weigh the benefits of cancer detection against the harms of overtreatment; however, the ideal age of screening onset and frequency remain uncertain. In 2020, the American Cancer Society (ACS) released new guidelines^22^ diverging from the 2012 consensus guidelines created by the ACS, US Preventive Services Task Force (USPSTF), and the American College of Obstetricians and Gynecologists (ACOG).^23^ The ACS proposed initiating screening at age 25 years rather than 21 years and preferentially testing for high-risk human papillomavirus (HPV) every 5 years over Papanicolaou testing every 3 years—changes that have been received with some controversy.^24,25,26^ Factors influencing the ACS’s recommendations include increasing rates of HPV vaccination, the US Food and Drug Administration’s approval of stand-alone high-risk HPV tests (which have higher sensitivity and negative predictive value than Papanicolaou tests), and concerns regarding potential overtreatment in young IWCs.^22^ In 2021, ACOG released guidelines that expanded options for IWCs aged 25 to 65 years to use high-risk HPV testing alone but were otherwise unchanged from previous guidelines.^26^

Establishing optimal parameters for screening frequency, age, and test type remains imperative, albeit challenging, given that much of the previous research in this area has been limited to small observational studies and clinical trials.^17,27^ In a large decision analysis, Kamphuis et al^28^ ran a simulated comparison of 8 cervical cancer screening approaches in various high-income countries to estimate their association with maternal and neonatal morbidity and mortality. The most intensive screening program was associated with an increase in maternal life years of 9%, a decrease in cervical cancer incidence of 67%, and a decrease in cervical cancer deaths of 75%, at the cost of 250% more preterm births compared with the least intensive program.

Herein, we ascertain the association between guidelines on the recommended number of cervical cancer screenings and downstream PTD risk using individual-level data on all US births to females aged 18 to 24 years. To our knowledge, this is the first population-level empirical analysis studying the association of cervical cancer screening policy with PTD risk. These estimates are essential for analyses weighing the costs and benefits of alternative cervical cancer screening strategies.

## Methods

### Study Population

The study population included US females aged 18 to 24 years. This age group was selected because their recommended number of screenings had the greatest variation between 1996 and 2018 (Figure; eFigure 1 in Supplement 1). This age group has also been shown to have the highest rates of spontaneous CIN grade 2 lesion regression (with 2-year regression rates estimated between 60% and 90%) and the highest rates of false-positive Papanicolaou test results (specificity estimates ranging between 64.8% and 76.5%), suggesting unnecessary excision procedures without added cervical cancer risk reduction.^8,12,29,30,31,32^ Additionally, IWCs younger than 24 years are less likely to have used assisted reproductive technologies prior to giving birth.^33^ Because we used deidentified publicly available data, this study was exempt from informed consent requirements and institutional review board review per the Common Rule. This study followed the Strengthening the Reporting of Observational Studies in Epidemiology (STROBE) reporting guideline.

**Figure.**
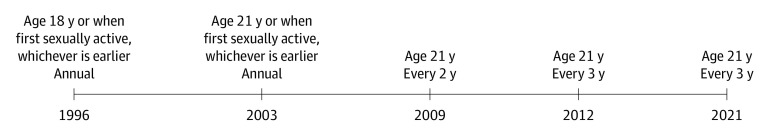
Evolution of the American College of Obstetricians and Gynecologists (ACOG) Cervical Cancer Screening Guidelines for Individuals With a Cervix Aged 18 to 24 Years From 1996 to 2021 A more comprehensive review comparing historical changes in the ACOG, American Cancer Society, and US Preventive Services Task Force guidelines is provided in eTable 2 in Supplement 1.

### Data Sources

We used data from the US Centers for Disease Control and Prevention’s National Center for Health Statistics (NCHS) to analyze associations between cervical cancer screening guidelines and birth outcomes. The National Vital Statistics System provided gestational age (GA) and maternal characteristics data from birth certificates for all births in the US from 1996 to 2018. We restricted analyses to singleton, nulliparous births delivered by females aged 18 to 24 years. We also used Behavioral Risk Factor Surveillance System (BRFSS) data in a supplementary analysis of the association between guidelines and actual screening adherence (eAppendix 1 in Supplement 1).

### Primary Outcomes

Primary outcomes were binary indicators for PTD and very preterm delivery (VPTD), defined as birth before 37 weeks’ and 34 weeks’ GA, respectively. We also examined GA, measured in weeks from the first day of the last menstrual period as reported on the birth certificate (eAppendix 2 in Supplement 1).

### Recommended Number of Cervical Cancer Screenings

We constructed a screenings exposure variable, the *recommended number of screenings*, representing how many screenings a female would have received based on her age and year she gave birth if she had followed the guidelines in place prior to giving birth (eTable 1 and eFigure 1 in Supplement 1). Using ACOG guidelines, we calculated the cumulative recommended number of screenings for each female in the sample based on age and year of childbirth. We chose to use the ACOG guidelines because gynecologists and primary care physicians reported these guidelines as being the most influential.^34^ The ACOG guidelines for IWCs aged 18 to 24 years are summarized in the Figure, which shows how major guideline changes led to variation in age of screening initiation and screening frequency over time. A comparison of ACOG, ACS, and USPSTF guideline changes over time is shown in eTable 2 in Supplement 1.

We treated the recommended number of screenings as a measure of the patient’s exposure to screening and the role of policy in changing that exposure over time. Ideally, we would have compared this variable with longitudinal data tracking the Papanicolaou testing history of every individual. However, such granular data have not been collected in a national database over the study period. Instead, we validated the recommended number of screenings using the best available data from the BRFSS on aggregate annual screening adherence rates (Papanicolaou test every 3 years [3-year Papanicolaou test] or any Papanicolaou test [ever Papanicolaou test]) (eFigures 4 and 5 in Supplement 1). We estimated a pairwise Pearson correlation between the recommended number of screenings and 3-year Papanicolaou test and ever Papanicolaou test rates (eAppendix 1 in Supplement 1). We also graphed trends in screening adherence between 1996 and 2018, overall and by race and ethnicity, to identify racial disparities in access to screenings throughout this period (eFigures 2 and 3 in Supplement 1).

### Control Variables

Maternal characteristics in the NCHS data included age, race and ethnicity, educational level, marital status, number of prenatal care visits, and maternal comorbidities. Maternal race and ethnicity were categorized as Black, Hispanic, White, or other race and ethnicity to enable standardization between BRFSS and NCHS data (specifically, NCHS data on race and ethnicity were collapsed into 4 groups to align with early BRFSS data categorization). The other race and ethnicity category included Alaska Native, American Indian, Asian, Pacific Islander, unknown or missing race or ethnicity, and multiracial. Educational attainment levels followed BRFSS categorization. Maternal comorbidities included maternal and gestational hypertension (combined as a single hypertension indicator due to the inability to disaggregate prior to 2000) and maternal and gestational diabetes (combined into a single diabetes indicator).

### Statistical Analysis

To analyze the association between the recommended number of cervical cancer screenings and PTD rates, we used a difference-in-differences analysis with a continuous treatment variable that leveraged variation in guidelines over time and across females of different ages. The primary β coefficient of interest was that on the recommended number of cervical cancer screenings variable, which represents the change in the probability of PTD associated with 1 additional guideline-recommended screening. We estimated a linear model with fixed effects for each year of age at childbirth, thus accounting for differences in PTD rates between females of different ages. The models also included birth year fixed effects to account for aggregate trends in PTD over time. We controlled for maternal educational level, race and ethnicity, comorbidities, marital status, and prenatal care visits. The analysis compared PTD rates among females of the same age who gave birth in different years and were recommended different numbers of cervical cancer screenings prior to delivery due to guideline changes over time, and PTD rates among females who gave birth in the same year but who were recommended to receive different numbers of screenings due to differences in age. Robust variance estimators were used to account for clustering on childbirth year and age. Further discussion of the model specification and supplemental analyses investigating heterogeneity in treatment effects over time and heterogeneous dynamic treatment effects across adoption cohorts are provided in eAppendix 3, eFigure 6, and eTable 4 in Supplement 1.^35^ We report 95% CIs from the original regression estimates in addition to Romano-Wolf corrected *P* values accounting for multiple hypothesis testing.^36^ A Wald test was used to evaluate whether the β coefficient on the recommended number of screenings variable for a given subgroup varied significantly from that of a specified reference subgroup. Finally, we conducted several subgroup analyses to determine the associations between the number of recommended cervical cancer screenings for different races and ethnicities and with and without hypertension and/or diabetes. We also conducted a supplemental analysis comparing females with and without potential HPV vaccine exposure based on maternal birth year (eAppendix 4 in Supplement 1). All data were analyzed between June 2020 and March 2023 using Stata, version 17 (StataCorp LLC).

## Results

A total of 11 333 151 females aged 18 to 24 years who had singleton, nulliparous births between 1996 and 2018 were included in the sample. Maternal characteristics and birth outcomes are presented in Table 1. Mean (SD) maternal age at childbirth was 20.9 (1.9) years, and the mean (SD) recommended number of cervical cancer screenings by the year of childbirth was 2.4 (2.2). Most mothers were White individuals (6 068 498 [53.5%]), followed by mothers of Hispanic (2 601 255 [23.0%]), Black (2 069 713 [18.3%]), and other race and ethnicity (593 715 [5.2%], including Alaska Native; American Indian; Asian; Pacific Islander; and unknown, missing race or ethnicity, or multiracial). Most individuals were unmarried (64.5%) and had a mean (SD) of 11.3 (3.8) prenatal care visits. A minority of mothers had diabetes (2.2%), hypertension (4.8%), or both comorbidities (0.3%). The median (IQR) GA was 39 (38-40) weeks, 1 140 490 (10.1%) of births were PTD, and 333 040 (2.9%) were VPTD. The mean (SD) recommended number of cervical cancer screenings in the sample decreased by 3 during the study period, from 3.8 (1.8) in 1996 to 0.8 (0.7) in 2018.

**Table 1.** Maternal Characteristics and Birth Outcomes

Characteristic	Value
Sample size	11 333 151
Maternal age, mean (SD), y	20.9 (1.9)
Estimated cervical cancer screenings, mean (SD)^a^	2.4 (2.2)
Race and ethnicity, No. (%)	
Black	2 069 713 (18.3)
Hispanic	2 601 225 (23.0)
White	6 068 498 (53.5)
Other^b^	593 715 (5.2)
Education, No. (%)	
Some high school	2 386 416 (21.1)
High school degree or some college	8 229 869 (72.6)
College degree or above	716 866 (6.3)
Marital status, No. (%)	
Married	7 306 073 (64.5)
Unmarried	4 027 078 (35.5)
Prenatal visits, mean (SD)	11.3 (3.8)
Maternal comorbidities, No. (%)	
Hypertension and/or diabetes^c^	766 001 (6.8)
Hypertension only^c^	540 278 (4.8)
Diabetes only^c^	254 883 (2.2)
Hypertension and diabetes^c^	27 360 (0.3)
Birth outcomes	
Gestational age, median (IQR), wk	39 (38-40)
Preterm birth before 37 wk, No. (%)^c^	1 140 490 (10.1)
Very preterm birth before 35 wk, No. (%)^c^	333 040 (2.9)

^a^ Variable was calculated from contemporaneous American College of Obstetricians and Gynecologists recommended cervical cancer screening guidelines.

^b^ Other race and ethnicity includes Alaska Native, American Indian, Asian, Pacific Islander, unknown or missing race or ethnicity, and multiracial.

^c^ Variables were calculated using original variables from the National Center for Health Statistics’ National Vital Statistics System.

### Association Between Recommended Number of Screenings and PTD

We found that recommended cervical cancer screening rates were directly associated with PTD and inversely associated with GA. We estimate that each additional recommended cervical cancer screening was associated with an increased risk of PTD (0.073 percentage points; 95% CI, 0.026 to 0.120), no significant change in VPTD risk (−0.00006 percentage points; 95% CI, −0.020 to 0.020 percentage points), and a younger GA (−0.016 weeks; 95% CI, −0.021 to −0.010 weeks) (Table 2 and eTable 3 in Supplement 1. Robustness analyses investigating heterogeneous treatment effects and potential bias from multiple treatment timings are included in eAppendix 3 and eFigure 6 in Supplement 1.

**Table 2.** Association Between Recommended Cervical Cancer Screenings and Preterm Delivery

	Estimate^a^
Preterm delivery (before 37 wk GA)	
Recommended No. of screenings, percentage-point difference (95% CI)^b^	0.073 (0.026 to 0.120)
Mean of dependent variable	0.10
Romano-Wolf *P* value	.001
Very preterm delivery (before 35 wk GA)	
Recommended No. of screenings, percentage-point difference (95% CI)^b^	−0.00006 (−0.020 to 0.020)
Mean of dependent variable	0.03
Romano-Wolf *P* value	.99
GA	
Recommended No. of screenings (95% CI), wk^c^	−0.016 (−0.021 to −0.010)
Mean of dependent variable	38.97
Romano-Wolf *P* value	.001

Abbreviation: GA, gestational age.

^a^ The SEs were clustered by mother’s age and birth year.

^b^ Reported as the percentage-point change in probability of preterm delivery per 1 additional recommended screening.

^c^ Reported as the change in GA (in weeks) per 1 additional recommended screening.

### Association Between Recommended Number of Screenings and PTD by Race and Ethnicity

For Black females, 1 additional recommended cervical cancer screening was associated with an increased PTD risk (0.120 percentage points; 95% CI, 0.029 to 0.210 percentage points) and younger GA (−0.012 weeks; 95% CI, −0.020 to −0.003 weeks). Among Hispanic females, we observed no statistically significant change in PTD risk, VPTD risk, or GA. For White females, 1 additional recommended screening was associated with an increased PTD risk (0.137 percentage points; 95% CI, 0.061 to 0.216 percentage points), increased VPTD risk (0.028 percentage points; 95% CI, 0.009 to 0.048 percentage points), and younger GA (−0.026 weeks; 95% CI, −0.035 to −0.017 weeks). Among females of other race or ethnicity, 1 additional recommended screening was associated with an increased PTD risk (0.218 percentage points; 95% CI, 0.103 to 0.333 percentage points) and younger GA (−0.020 weeks; 95% CI, −0.031 to −0.010 weeks). Results of Wald tests showed that this change in PTD risk relative to White females varied across all groups (Table 3).

**Table 3.** Association Between Recommended Cervical Cancer Screenings and Preterm Delivery by Race and Ethnicity^a^

	Race and ethnicity			
	Black females	Hispanic females	White females	Other females^b^
Preterm delivery (before 37 wk GA)				
Recommended No. of screenings, percentage-point difference (95% CI)^c^	0.120 (0.029 to 0.210)	−0.068 (−0.137 to 0.002)	0.137 (0.061 to 0.213)	0.218 (0.103 to 0.333)
Mean of dependent variable	0.14	0.10	0.09	0.10
Romano-Wolf *P* value	.049	.14	.02	.03
Very preterm delivery (before 34 wk GA)				
Recommended No. of screenings, percentage-point difference (95% CI)^c^	0.039 (−0.016 to 0.094)	−0.049 (−0.085 to −0.013)	0.028 (0.009 to 0.048)	0.012 (−0.021 to 0.081)
Mean of dependent variable	0.0482	0.0269	0.0242	0.0278
Romano-Wolf *P* value	.18	.07	.02	.30
GA				
Recommended No. of screenings (95% CI), wk^d^	−0.012 (−0.020 to −0.003)	0.002 (−0.001 to 0.006)	−0.026 (−0.035 to −0.017)	−0.020 (−0.031 to −0.010)^b^
Mean of dependent variable	38.55	38.98	39.11	38.94
Romano-Wolf *P* value	.03	.23	.001	.03
Wald test *P* value^e^	.001	.001	NA	.009

Abbreviation: GA, gestational age; NA, not applicable.

^a^ The SEs were clustered by mother’s age and birth year.

^b^ Other race and ethnicity includes Alaska Native, American Indian, Asian, and Pacific Islander females, females of unknown or missing race or ethnicity, and multiracial females.

^c^ Reported as the percentage-point change in probability of preterm delivery per 1 additional recommended screening.

^d^ Reported as the change in GA (in weeks) per 1 additional recommended screening.

^e^ To test for heterogeneity across groups, we regressed preterm delivery risk on screenings (and all controls) linearly interacted with the race and ethnicity indicators. The null hypothesis that there was no difference in the change in preterm delivery risk associated with 1 additional screening for a given race or ethnicity subgroup compared with White females was rejected for Black, Hispanic, and other race groups at the level of α = .01.

### Association Between Recommended Number of Screenings and PTD by Comorbidities and Maternal HPV Exposure

Among females with comorbidities, 1 additional cervical cancer screening was associated with an increased PTD risk (0.255 percentage points; 95% CI, 0.109 to 0.400 percentage points) and younger GA (−0.023 weeks; 95% CI, −0.035 to −0.011 weeks) (Table 4). Females without diabetes or hypertension had an increased PTD risk (0.059 percentage points; 95% CI, 0.014-0.103 percentage points) and younger GA (−0.015 weeks; 95% CI, −0.020 to −0.010 weeks). These differences in PTD risk, as assessed using the Wald test, varied between females with vs without diabetes or hypertension (Table 4).

**Table 4.** Association Between Recommended Cervical Cancer Screenings and Preterm Delivery (PTD) by Maternal Comorbidity Status^a^

	Females with hypertension and/or diabetes (n = 766 001)	Females without hypertension or diabetes (n = 10 567 150)
Preterm delivery (before 37 wk GA)		
Recommended No. of screenings, percentage-point difference (95% CI)^b^	0.255 (0.109 to 0.400)	0.059 (0.014 to 0.103)
Mean of dependent variable	0.18	0.10
Romano-Wolf *P* value	.001	.04
Very preterm delivery (before 34 wk GA)		
Recommended No. of screenings, percentage-point difference (95% CI)^b^	0.011 (−0.068 to 0.088)	−0.002 (−0.020 to 0.015)
Mean of dependent variable	0.06	0.03
Romano-Wolf *P* value	.78	.77
GA		
Recommended No. of screenings (95% CI), wk^c^	−0.023 (−0.035 to −0.011)	−0.015 (−0.020 to −0.010)
Mean of dependent variable	38.22	39.02
Romano-Wolf *P* value	.001	.001
Wald test *P* value^d^	<.001	NA

Abbreviations: GA, gestational age; NA, not applicable.

^a^ The SEs were clustered by mother’s age and birth year.

^b^ Reported as the percentage-point change in probability of PTD per 1 additional recommended screening.

^c^ Reported as the change in GA (in weeks) per 1 additional recommended screening.

^d^ To test for heterogeneous effect sizes for females with and without common comorbidities, we regressed the PTD indicator on Recommended Number of Screenings (and all controls) linearly interacted with the hypertension and/or diabetes indicator. The null hypothesis that the associated change in PTD risk from screenings was equal for females with and without comorbidities was rejected at the level of α = .001.

Results from a supplemental subgroup analysis for potential maternal HPV vaccine exposure are reported and discussed in eTable 4 and eAppendix 4 in Supplement 1.

## Discussion

Results of this study suggest that higher rates of cervical cancer screening are associated with an increased risk of PTD among young females. Specifically, 1 additional recommended screening before childbirth was associated with an increase in PTD absolute risk of 0.073 (95% CI, 0.026-0.120) percentage points or a relative increase in risk of 0.73% when evaluated at the mean of the dependent variable. One interpretation of this coefficient could be in terms of additive risk for a population of 100 000 IWCs. This estimate suggests that we could anticipate 73 additional PTDs per 100 000 females for every 1 additional recommended screening before childbirth. Therefore, in the terminal study year (2018) an estimated 1348 PTDs could have been averted (3% relative reduction) due to reduced screening requirements (eAppendix 5 in Supplement 1).

The Kamphuis et al^28^ simulation reported 28 additional PTDs per 100 000 females associated with cervical cancer screening every 3 years starting at age 21 years instead of 24 years, and an additional 112 PTDs per 100 000 females associated with screening starting at age 21 years instead of 30 years. The effect size in the present study was larger than the estimate by Kamphuis et al,^28^ empirically supporting that the increased PTD risk associated with additional cervical cancer screenings is at least as large as their simulated estimate. A full analysis of the risks and benefits of incremental screening is necessary to evaluate the trade-off with increased cervical cancer mortality. We believe our results could most appropriately be used as inputs into a new decision analysis model with maternal and neonatal outcome trade-offs for future comparisons of guideline programs. The larger effect size found in the present study may reflect that the analysis was focused on young females, who are more likely to have false-positive results on Papanicolaou tests and spontaneous lesion regression and to undergo unnecessary treatment.^9,12,37,38^

This analysis focused on the association between recommended cervical cancer screening guidelines and PTD and not on the association between actual screenings received and PTD. Given that compliance with guidelines is unlikely to be 100%, the implications of actual screenings for PTD risk may be larger. For any given guideline change, if compliance were 80% (the approximate maximum 3-year Papanicolaou testing rate reported in BRFSS data), an increase of 1 actual screening could mean 91 additional PTDs per 100 000 females.

### Subgroups at Higher Risk of PTD

We found that the adjusted risk of PTD associated with changes in screening recommendations was higher in females with hypertension or diabetes compared with females without either comorbidity. The mechanism underlying these differences is not known, although these comorbidities may increase the risk for cervical stress prior to or during pregnancy.^39,40,41^ Additional research is needed to better understand this association and if differential screening strategies might be beneficial for females at higher risk of PTD.

Effect sizes also varied by race and ethnicity. White females and females of other race or ethnicity had the largest increases in PTD risk with 1 additional recommended screening. Black females had a smaller increase in PTD risk compared with White females. Surprisingly, among Hispanic females, there was no significant association between recommended cervical cancer screenings and PTD risk. This finding may be consistent with previous work documenting better birth outcomes among Hispanic individuals for reasons poorly understood.^42^ Results of the present study suggest that the shift toward less frequent cervical cancer screenings was associated with better outcomes for Alaska Native, American Indian, Asian, Pacific Islander, White, and multiracial females, which stands to not reduce, but rather potentially exacerbate, racial disparities in birth outcomes by generating the largest improvements in birth outcomes among White individuals who already have the lowest rates of poor outcomes.

### Future Directions

An area for future research is the association between PTD risk and maternal HPV vaccine exposure; our analysis of the topic and conclusions were extremely limited given our lack of data on actual vaccination status (eAppendix 4 and eTable 4 in Supplement 1). As the proportion of vaccinated individuals who have reached screening-eligible and reproductive age increases, there may be an opportunity to safely delay the initiation of cervical cancer screening (as proposed by the ACS).^22^ However, racial and ethnic disparities have been observed in vaccine uptake, which may contribute not only to continuing disparities in HPV-related cancers but also to disparities in cervical cancer screening necessity and downstream implications for PTD risk.^43^

Finally, results of the present study support the broader finding that prior cervical procedures are a key risk factor for PTD.^3,4,5,13,14,15,16,17^ These findings highlight the need for more research on less invasive techniques to biopsy suspicious lesions, the use of cerclage and other interventions to reduce PTD risk, and potential opportunities for cervical cancer treatment guidelines to also incorporate childbearing plans into procedural timing consideration.

### Limitations

This study has limitations. First, it lacks data on actual Papanicolaou test history among females giving birth in the NCHS data. We addressed this gap by validating the recommended number of screenings variable using BRFSS data on Papanicolaou screening prevalence. Second, we used recommended cervical cancer screenings rather than actual screening in interpreting our estimates. Third, we offered an empirical estimate of the PTD risks associated with screening guidelines but did not have the data necessary to evaluate reciprocal implications for cervical cancer mortality. Therefore, these findings alone cannot identify the optimal screening strategy but may be used to inform future decision analyses toward this objective. Fourth, our results apply only to females aged 18 to 24 years; additional studies are needed to extend these findings to older females for whom additional confounders, such as assisted reproductive therapies, may become a larger concern. Fifth, US states varied in what race and ethnicity data they collected between 1996 and 2018, and the data were not presented in standardized groups until 2005. To use consistent race and ethnicity groups across the study period, we collapsed the data into 4 groups aligned with early BRFSS data categorization. While the findings suggest that differences in the effect sizes by races and ethnicities may exist, this association was difficult to assess given the crude quality of demographic data. Sixth, the demographic data available include only female sex and do not differentiate by gender (eg, transgender men, nonbinary IWCs, cisgender women). Future survey collection should monitor outcomes among gender-minority IWCs to better care for these underserved populations.

## Conclusion

This cross-sectional study found that an increasing number of recommended cervical cancer screenings was associated with an increased risk of PTD among females aged 18 to 24 years, especially for females with hypertension or diabetes. Although based on US data, results from this study may be helpful for public health entities outside of the US, particularly in countries where the prevalence of cervical cancer is considerably higher and Papanicolaou screenings continue to play a major role in reducing cervical cancer incidence and mortality. Overall, this study may help inform future recommendations for screening practices by furthering our understanding of the trade-offs involved in terms of maternal and neonatal outcomes.

## Notes

Supplement 1. **eAppendix 1.** Supplemental Analysis of Recommended Number of Screenings and Actual Screening Rates **eAppendix 2.** Gestational Age Estimate **eAppendix 3.** Difference-in-Differences Estimation Assumptions and Extensions **eAppendix 4.** Potential Maternal HPV Vaccine Exposure Subanalysis **eAppendix 5.** PTDs Averted in 2018 Due to Reduced Recommended Screenings **eTable 1.** Recommended Number of Screenings by Age at Birth by Childbirth Year **eTable 2.** History of Cervical Cancer Screenings from ACOG, ACS, and USPSTF from 1950-2021 **eTable 3.** Effect of Cervical Cancer Screenings on Neonatal Outcomes, Extended Table with Coefficients **eTable 4.** HPV Exposure Subgroup Analysis **eFigure 1.** Recommended Number Of Screenings by Age at Birth between 1996-2018 **eFigure 2.** Cervical Cancer Screening Trends from 1996-2018 by Age, Ever-Pap and 3-Year Pap Test Rates **eFigure 3.** Cervical Cancer Screening Trends from 1996-2018 by Race/Ethnicity, Ever-Pap and 3-Year Pap Tests **eFigure 4.** BRFSS Screening Data Correlation with Recommended Screening Variable, Ever-Pap and 3-Year Pap Tests **eFigure 5.** BRFSS Screening Data Correlations with Recommended Screening Variable by Race and Ethnicity, Ever-Pap and 3-Year Pap **eFigure 6.** Difference-in-Differences with Multiple Treatment Timings Event Study Graph
eReferences

Supplement 2.
Data Sharing Statement
