# Supplementary material for: Association Between Cervical Cancer Screening Guidelines and Preterm Delivery Among Females Aged 18 to 24 Years
Source: JAMA Health Forum. 2023 Jul 21;4(7):e231974. doi: 10.1001/jamahealthforum.2023.1974 (PMC10362467; doi:10.1001/jamahealthforum.2023.1974)
Supplement: Supplement 1. — eAppendix 1. Supplemental Analysis of Recommended Number of Screenings and Actual Screening Rates eAppendix 2. Gestational Age Estimate eAppendix 3. Difference-in-Differences Estimation Assumptions and Extensions eAppendix 4. Potential Maternal HPV Vaccine Exposure Subanalysis eAppendix 5. PTDs Averted in 2018 Due to Reduced Recommended Screenings eTable 1. Recommended Number of Screenings by Age at Birth by Childbirth Year eTable 2. History of Cervical Cancer Screenings from ACOG, ACS, and USPSTF from 1950-2021 eTable 3. Effect of Cervical Cancer Screenings on Neonatal Outcomes, Extended Table with Coefficients eTable 4. HPV Exposure Subgroup Analysis eFigure 1. Recommended Number Of Screenings by Age at Birth between 1996-2018 eFigure 2. Cervical Cancer Screening Trends from 1996-2018 by Age, Ever-Pap and 3-Year Pap Test Rates eFigure 3. Cervical Cancer Screening Trends from 1996-2018 by Race/Ethnicity, Ever-Pap and 3-Year Pap Tests eFigure 4. BRFSS Screening Data Correlation with Recommended Screening Variable, Ever-Pap and 3-Year Pap Tests eFigure 5. BRFSS Screening Data Correlations with Recommended Screening Variable by Race and Ethnicity, Ever-Pap and 3-Year Pap eFigure 6. Difference-in-Differences with Multiple Treatment Timings Event Study Graph eReferences [file jamahealthforum-e231974-s001.pdf]

Supplemental Online Content

Bromley-Dulfano RA, Rossin-Slater M, Bundorf MK. Association between cervical cancer screening guidelines and preterm delivery among females aged 18 to 24 years. *JAMA Health Forum.* 2023;4(7):e231974.
doi:10.1001/jamahealthforum.2023.1974

eAppendix 1. Supplemental Analysis of Recommended Number of Screenings and Actual Screening Rates

eAppendix 2. Gestational Age Estimate

eAppendix 3. Difference-in-Differences Estimation Assumptions and Extensions

eAppendix 4. Potential Maternal HPV Vaccine Exposure Subanalysis

eAppendix 5. PTDs Averted in 2018 Due to Reduced Recommended Screenings

eTable 1. Recommended Number of Screenings by Age at Birth by Childbirth Year

eTable 2. History of Cervical Cancer Screenings from ACOG, ACS, and USPSTF from 1950-2021

eTable 3. Effect of Cervical Cancer Screenings on Neonatal Outcomes, Extended Table with Coefficients

eTable 4. HPV Exposure Subgroup Analysis

eFigure 1. Recommended Number Of Screenings by Age at Birth between 1996-2018

eFigure 2. Cervical Cancer Screening Trends from 1996-2018 by Age, Ever-Pap and 3-Year Pap Test Rates

eFigure 3. Cervical Cancer Screening Trends from 1996-2018 by Race and Ethnicity, Ever-Pap and 3-Year Pap Tests

eFigure 4. BRFSS Screening Data Correlation with Recommended Screening Variable, Ever-Pap and 3-Year Pap Tests

eFigure 5. BRFSS Screening Data Correlations with Recommended Screening Variable by Race and Ethnicity, Ever-Pap and 3-Year Pap

eFigure 6. Difference-in-Differences with Multiple Treatment Timings Event Study Graph

eReferences

This supplemental material has been provided by the authors to give readers additional information about their work.

eAppendix 1: Supplemental Analysis of Recommended Number of Screenings and Actual Screening Rates

Data Sources and Analysis

Behavioral Risk Factor Surveillance System (BRFSS) data from the Centers for Disease Control and Prevention is a nationally representative survey with information on cervical cancer screening rates from 1996 to 2010 and even years from 2012 to 2018. We focused on two measures of adherence: an indicator for having a Pap test in the past three years (3-year Pap), and an indicator for having ever received a Pap test (ever-Pap). We also obtained age, education, and race and ethnicity data from the BRFSS.

Relationship between the Recommended Number of Screenings and Actual Screening Rates

Both 3-year Pap and ever-Pap rates were relatively constant from 1996 to 2002 and then decreased between 2003 and 2018 (Supplemental eFigure 2). Ever-Pap rates declined from 84% in 1996 to 51% in 2018. 3-year Pap rates decreased from 81% to 48%. The decrease was mainly driven by declines in screenings among 18 to 20-year-olds. This is consistent with the changes in screening guidelines over this period which recommended delaying the age of screening onset to 21. Ever-Pap and 3-year Pap rates increased with age. Black and White women tended to have higher screening rates, while Hispanic women tended to have lower rates (Supplemental eFigure 3).

There was strong correlation between BRFSS reported screening rates and the Recommended Number of Screenings variables for both ever-Pap and 3-year Pap measures (Pearson correlation coefficients 0.88 and 0.89 respectively; Supplemental eFigure 4). Similarly high degrees of correlation were observed across different race and ethnicity groups (Supplemental eFigure 5). Because actual adherence is typically lower than guideline recommendations, the Recommended Number of Screenings likely represents an overestimate of the number of screenings an individual woman received.

eAppendix 2: Gestational Age Estimate

We opted to use the Last Menstrual Period (LMP) definition for defining gestational age because it was consistent over time across the duration of our study period. It is important to note that this definition is not the current standard used by the CDC. The obstetric estimate has been the standard since 2014 and the LMP definition has been shown to result in higher rates of PTD. However, previous literature including the CDC report above shows a high degree of correlation reported between the two measures. While the LMP-based definition may represent an overestimation of preterm birth rates, there is a corresponding documentation of underestimation of neonatal mortality for infants delivered preterm. A thorough comparison of the two measures is available from the CDC: https://www.cdc.gov/nchs/data/nvsr/nvsr64/nvsr64_05.pdf. The obstetric estimate is preferred for current use and should be used for future research in this area as possible.

eAppendix 3: Difference-in-Differences Estimation Assumptions and Extensions

A key assumption underlying causal identification from DiD models is an assumption of parallel trends in untreated outcomes, or in other words, that in the absence of treatment, the difference between the 'treatment' and ‘control’ group would be constant over time. Recent literature suggests that standard two-way fixed effects DiD models with multiple treatment periods may be biased due to variation in treatment timing and potentially heterogeneous treatment effects over time. In our analysis, the bias would occur if the impact of recommended screenings affected PTD risk differentially in different years, and if the treatment effect itself varied over time. Several recent methods have been developed to relax the standard “parallel trends” assumption and address the issues stemming from variation in treatment timing.[1,2] We augment our analysis with an estimation using a recent method developed by Callaway and Sant’Anna (2021) which addresses these issues. This approach relies on having a binary treatment indicator, and thus we first transform our Recommended Number of Screenings variable accordingly.

Specifically, we create a binary indicator that is equal to 1 if a mother is recommended fewer than 2 screenings by the time of childbirth, and 0 otherwise. Thus, in this analysis we are studying the relationship between having fewer than 2 recommended screenings and the risk of PTD. Given that our baseline analysis found that an additional screening was associated with a higher risk of PTD, we expect to see that having fewer than 2 screenings (as opposed to 2 or more) is associated with a lower risk of PTD.

The results of this model using techniques developed by Callaway-Sant'Anna with the same controls from our main model yielded an average treatment effect on the treated (ATT) of -0.063 pp (95% CI: 0.097, -0.222, p-value 0.184), shown in Supplemental eFigure 6. The event-study figure provides evidence against pre-trends of concern and qualitatively supports that being recommended fewer than 2 screenings reduces the likelihood of PTD, in alignment with our baseline result that being recommended higher numbers of screenings increases the likelihood of PTD.

eAppendix 4: Potential Maternal HPV Vaccine Exposure Subanalysis

To investigate whether Maternal HPV Vaccine exposure might mediate any of the effects, we conducted a subgroup analysis between the cohort of females who were ever eligible for (and therefore potentially exposed to) the HPV vaccine. The first-generation Gardasil vaccine gained FDA approval in 2006 and beginning in 2007 was recommended for children aged 11 and 12. Gardasil 9 was approved by the FDA in 2014 and has gradually become the only available option in the US. Various schedules for missed and catch-up vaccination recommendations for individuals 13 and older have been proposed. Initial schedules proposed catch-up vaccinations up to age 18, then later iterations gradually relaxed the upper limit to age 26, and currently allow for catch-up vaccinations to age 45 upon physician-patient discretion.

To model potential HPV vaccine exposure, we include a dummy variable for mothers who were born in 1989 or later (or in other words mothers who would have been 11-18 years old from 2007 onwards and therefore eligible for potential vaccination). Our results, presented in Supplemental eTable 4, show that effects may be stronger for younger cohorts (i.e., those likely exposed to the HPV vaccine), though the estimates are not statistically significant. However, because HPV vaccine uptake remained very low for several years following its rollout (and was found to have significant racial disparities in its dissemination and uptake), it is also possible the trends are a reflection of heterogeneity along the many other dimensions by which cohorts differ over time.[3] Because of this major limitation, we are hesitant to draw conclusions about the role of vaccine exposure from this limited preliminary analysis.

eAppendix 5: PTDs Averted in 2018 Due to Reduced Recommended Screenings

The number of PTDs averted in 2018 were calculated by multiplying the number of births in 2018 times the decrease in recommended screenings between 1996 to 2018 times the coefficient for increased PTD per additional screen (615,770 births in 2018 * 3 fewer average screenings from 1996 to 2018 * 0.00073 PTD risk/recommended screen=1,348 preterm births averted in 2018).

This means in the absence of the guidelines changing from 1996, we would have expected 1,348 additional PTDs on top of the 43,227 observed PTDs in 2018 (1,348 + 43,227=44,575 PTDs). Together, this suggests a relative reduction of 1 - (43,227/44,575) = 3% fewer PTDs in 2018 due to reduced screening requirements.

eTable 1. Recommended Number of Screens by Age at Birth by Childbirth Year

This table details the recommended number of cumulative screenings that individuals of different ages were recommended to receive in different years according to contemporaneous guidelines of the year shown. Highlighted rows represent years of major ACOG guideline changes. The model assumes a one-year policy adoption lag after 2003, 2009, and 2012 policy changes.

	**18**	**19**	**20**	**21**	**22**	**23**	**24**
**1996**	1	2	3	4	5	6	7
**1997**	1	2	3	4	5	6	7
**1998**	1	2	3	4	5	6	7
**1999**	1	2	3	4	5	6	7
**2000**	1	2	3	4	5	6	7
**2001**	1	2	3	4	5	6	7
**2002**	1	2	3	4	5	6	7
**2003**	1	2	3	4	5	6	7
**2004**	0	1	2	4	5	6	7
**2005**	0	0	1	3	5	6	7
**2006**	0	0	0	2	4	6	7
**2007**	0	0	0	1	3	5	7
**2008**	0	0	0	1	2	4	6
**2009**	0	0	0	1	2	3	5
**2010**	0	0	0	1	1	2	3
**2011**	0	0	0	1	1	2	3
**2012**	0	0	0	1	1	2	2
**2013**	0	0	0	1	1	1	2
**2014**	0	0	0	1	1	1	2
**2015**	0	0	0	1	1	1	2
**2016**	0	0	0	1	1	1	2
**2017**	0	0	0	1	1	1	2
**2018**	0	0	0	1	1	1	2

eTable 2. History of Cervical Cancer Screenings from ACOG, ACS, and USPSTF from 1950-2021

	ACOG	ACS	USPSTF
1950s-1980			
	Annual screenings starting at age 18.	Annual screenings starting at age 18.	
1980			
	Age 18-65: Recommend annual screening.	Age 20-65: Pap test annually until 2 negative examinations. After this, Pap test at least every 3 years until age 65.<br>Over 65: end screening	USPSTF Formed in 1984
1987			
	ACOG and ACS Consensus statement:<br>Age 18-65: Recommend annual screening Pap test.<br><br>After at least 3 annual consecutive normal Pap tests, the screening interval could be extended at the physician's discretion.	Agreed to Consensus Statement.	
Additional notes	Data begin implicating HPV as the likely oncogenic agent in cervical cancer. The ACS, therefore, considered any woman who had ever had sexual intercourse to be at risk. Pap test also recognized to be served as a vehicle for many women to get other preventive health services.		
1995			
	Revised recommendation for women to return to annual screening (recommending against the extension of the screening interval based on physician discretion)		
2002-2003	2003	2002	2003
Start	Age 21-29: begin annual screenings at 21 years of age or onset of vaginal intercourse.<br><br>Age 30-70: At or after age 30, women who have had three consecutive, normal/ negative cytology results may be screened every two to three years	Age 21-29: begin annual screenings at 21 years of age or onset of vaginal intercourse.<br><br>Age 30-70: At or after age 30, women who have had three consecutive, normal/ negative cytology results may be screened every two to three years	Age 21-65: begin within 3 years of onset of sexual activity or age 21 (whichever comes first). Annual screening is still permitted but is not indicated as preferable.<br><br>Over 65: end screening.

	Over 70: end screening	Over 70: end screening	
HPV		"HPV DNA testing with cytology for primary cervical cancer screening has not been approved by the FDA. Based on the available data, both published and unpublished, the ACS guideline review panel found this technology to be promising. Should the FDA approve HPV DNA testing for this purpose, it would be reasonable to consider that for women aged 30 and over, as an alternative to cervical cytology testing alone, cervical screening may be performed every three years using conventional or liquid-based cytology combined with a test for DNA from high-risk HPV types." -ACS, 2002	
2007-2009	2009	2007	
Cytology Screening	Age 21-29: Pap test every 2 years for women aged 21-29 years<br>Age 30-65: after three consecutive normal Pap tests, screen every 3 years		
HPV Vaccination		Age 11-12: Routine HPV vaccination is recommended for females aged 11 to 12 years.<br>Age 13-18: HPV vaccination is also recommended to catch up on missed doses.	
2012			
Cytology Screening Alignment	Age 21-29: Pap test every 3 years<br>Age 30-65: Pap test every 3 years or HPV/Pap cotest every 5 years<br>Over 65: No screening if a series of prior tests were normal	Age 21-29: Pap test every 3 years<br>Age 30-65: HPV/Pap cotest every 3 years (preferred)<br>Pap test every 3 years (acceptable)<br>Over 65: No screening if a series of prior tests were normal	Age 21-29: Pap test every 3 years<br>Age 30-65: Pap test every 3 years, HPV test every 5 years, or HPV/Pap cotest every<br>5 years<br>Over 65: No screening if a series of prior tests were normal
HPV		HPV testing alone not recommended in any age group	
2018-2021	2021	2020	2018
	Age 21-29: Pap test alone every 3 years. HPV testing alone can be considered for women who are 25 to 29, but Pap tests are preferred.<br><br>Age 30-65: Choose between: co-testing every 5 years, Pap test alone	Age 21-24:No screening<br><br>Age 24-29: HPV test every 5 years (preferred), HPV/Pap cotest every 5 years (acceptable), OR Pap test every 3 years (acceptable)	Age 21-29: Pap test every 3 years<br><br>Age 30–65: Pap test every 3 years, HPV test every 5 years, or HPV/Pap cotest every<br>5 years

	every 3 years, HPV test alone every 5 years<br><br>Over 65: No screening if a series of prior tests were normal and not at high risk for cervical cancer	Age 30–65: HPV test every 5 years (preferred), HPV/Pap cotest every 5 years (acceptable), OR Pap test every 3 years (acceptable)<br><br>Over 65: No screening if a series of prior tests were normal	Over 65: No screening if a series of prior tests were normal and not at high risk for cervical cancer

eTable 3. Effect of Cervical Cancer Screenings on Neonatal Outcomes, Extended Table with Coefficients

Controls included individual mother's age, birth year, mother's marital status, number of maternal prenatal visits, maternal hypertension, maternal diabetes, mother's race, and mother's education. Number of recommended screenings refers to the estimated number of recommended Pap Tests that an individual should have received based on age and childbirth year, assuming they had followed the guidelines in place prior to giving birth. Standard errors were clustered by mother's age and childbirth year [95% CIs listed in brackets]. *p<0.05, **p<0.01, ***p<0.001

	**PTD (<37 weeks)**	**VPTD (<34 weeks)**	**Gestational Age (weeks)**
Recommended No. of Screenings	0.000729**	-0.000000617	-0.0155***
	[0.000258,0.00120]	[-0.000177,0.000175]	[-0.0207,-0.0103]
Mother is Married	-0.0102***	-0.00507***	0.0208*
	[-0.0115,-0.00889]	[-0.00543,-0.00471]	[0.00399,0.0375]
No. of Maternal Prenatal Visits	-0.0118***	-0.00638***	0.123***
	[-0.0121,-0.0115]	[-0.00649,-0.00627]	[0.121,0.126]
Mother has Hypertension	0.106***	0.0376***	-0.979***
	[0.102,0.111]	[0.0358,0.0394]	[-1.006,-0.952]
Mother has Diabetes	0.0604***	0.0144***	-0.616***
	[0.0559,0.0649]	[0.0128,0.0159]	[-0.657,-0.576]
**Mother's Race and Ethnicity**			
Black	0.0327***	0.0173***	-0.454***
	[0.0303,0.0351]	[0.0160,0.0186]	[-0.478,-0.430]
Hispanic	-0.00251**	-0.00268***	-0.0449**
	[-0.00389,-0.00113]	[-0.00319,-0.00217]	[-0.0700,-0.0199]
White	0	0	0
	[0,0]	[0,0]	[0,0]
Other	0.00117	-0.00192***	-0.0562***
	[-0.0000811,0.00241]	[-0.00250,-0.00134]	[-0.0749,-0.0375]
**Mother's Education**			
Some High School	0	0	0
	[0,0]	[0,0]	[0,0]
High School Degree or Some College	-0.00211***	0.000637**	-0.0740***
	[-0.00290,-0.00132]	[0.000248,0.00103]	[-0.0811,-0.0669]
College Degree or Above	-0.0133***	-0.00428***	-0.0360***
	[-0.0148,-0.0118]	[-0.00490,-0.00366]	[-0.0544,-0.0175]
Dep. var mean	0.101	0.0294	38.97
N	11333151	11333151	11333151

eTable 4. HPV Exposure Subgroup Analysis

This subgroup analysis compared mothers who gave birth before 1989 and therefore were unlikely to have received the HPV vaccine and mother's born later who plausibly could have been exposed to the vaccine. Controls included individual mother's age, birth year, mother's marital status, number of maternal prenatal visits, maternal hypertension, maternal diabetes, mother's race, and mother's education. Number of recommended screenings refers to the estimated number of recommended Pap tests that an individual should have received based on age and childbirth year, assuming they had followed the guidelines in place prior to giving birth. Standard errors were clustered by mother's age and childbirth year [95% CIs listed in brackets]. *p<0.05, **p<0.01, ***p<0.001

[a] Reported as the percentage point change in probability of preterm delivery per 1 additional recommended screening

[b] Reported as the change in GA (in weeks) per 1 additional recommended screening

		**Possible HPV Vaccine Exposure (Mother born in 1989 or later, N=3986880)**	**Less likely HPV Vaccine Exposure (Mother born before 1989, N=7346271)**
**Preterm Delivery (<37 weeks)**	Recommended No. of Screenings, percentage-point difference[a]	0.038	-0.0692
	95% CI	[-0.0221,0.0981]	[-0.171,0.0328]
	Dep. var mean	0.0989	0.102
**Very Preterm Delivery (<34 weeks)**	Recommended No. of Screenings, percentage-point difference[a]	0.0423	-0.0138
	95% CI	[-0.0139,0.0984]	[-0.000594,0.0318]
	Dep. var mean	0.0295	0.0293
**Gestational Age (Weeks)**	Recommended No. of Screenings, wk[b]	-0.0115**	0.00676
	95% CI	[-0.0182,-0.00475]	[-0.00128,0.0148]
	Dep. var mean	38.9	39.01

eFigure 1. Recommended Number Of Screenings by Age at Birth between 1996-2018. The figure details the recommended number of cumulative screenings that an individual would have received based on their age and year of childbirth assuming they had followed the guidelines in place prior to giving birth. Dashed vertical lines represent years of major ACOG guideline changes.

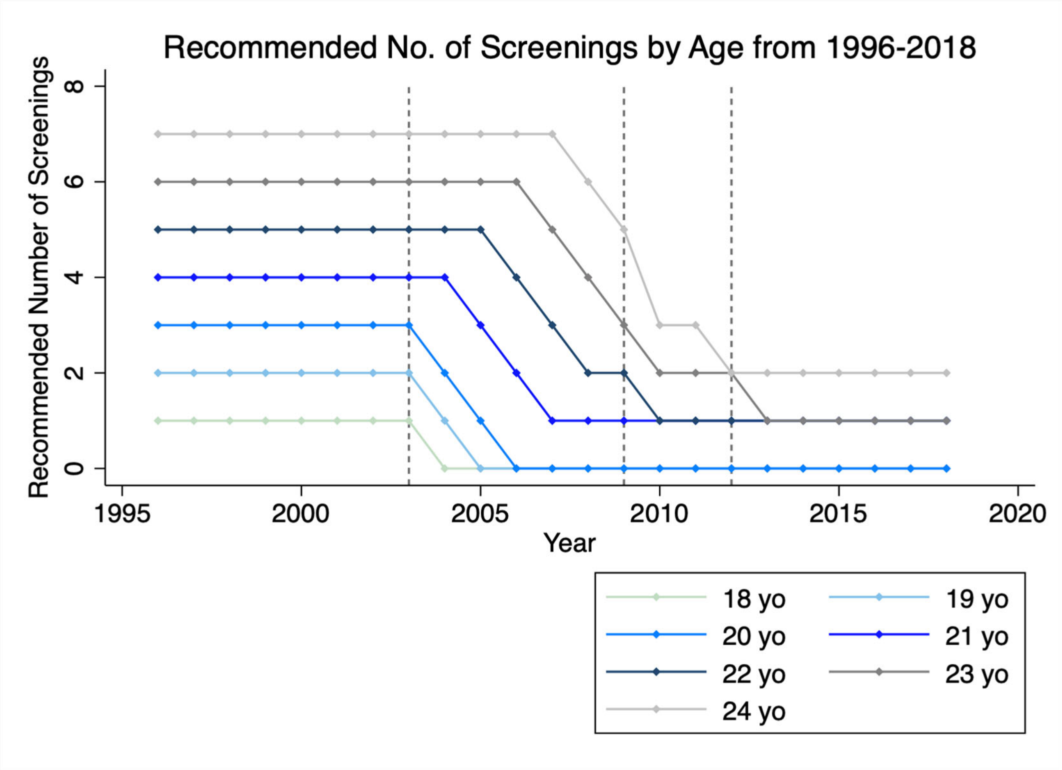

eFigure 2. Cervical Cancer Screening Trends from 1996-2018 by Age, Ever-Pap and 3-Year Pap Test Rates. The figure shows screening rates from BRFSS data representing the percentage of respondents who reported ever having received a Pap test (Ever-Pap, top panel) or receiving a Pap test in the past three years (3-Year Pap, bottom panel) by age. Dashed vertical lines represent years of major ACOG guideline changes.

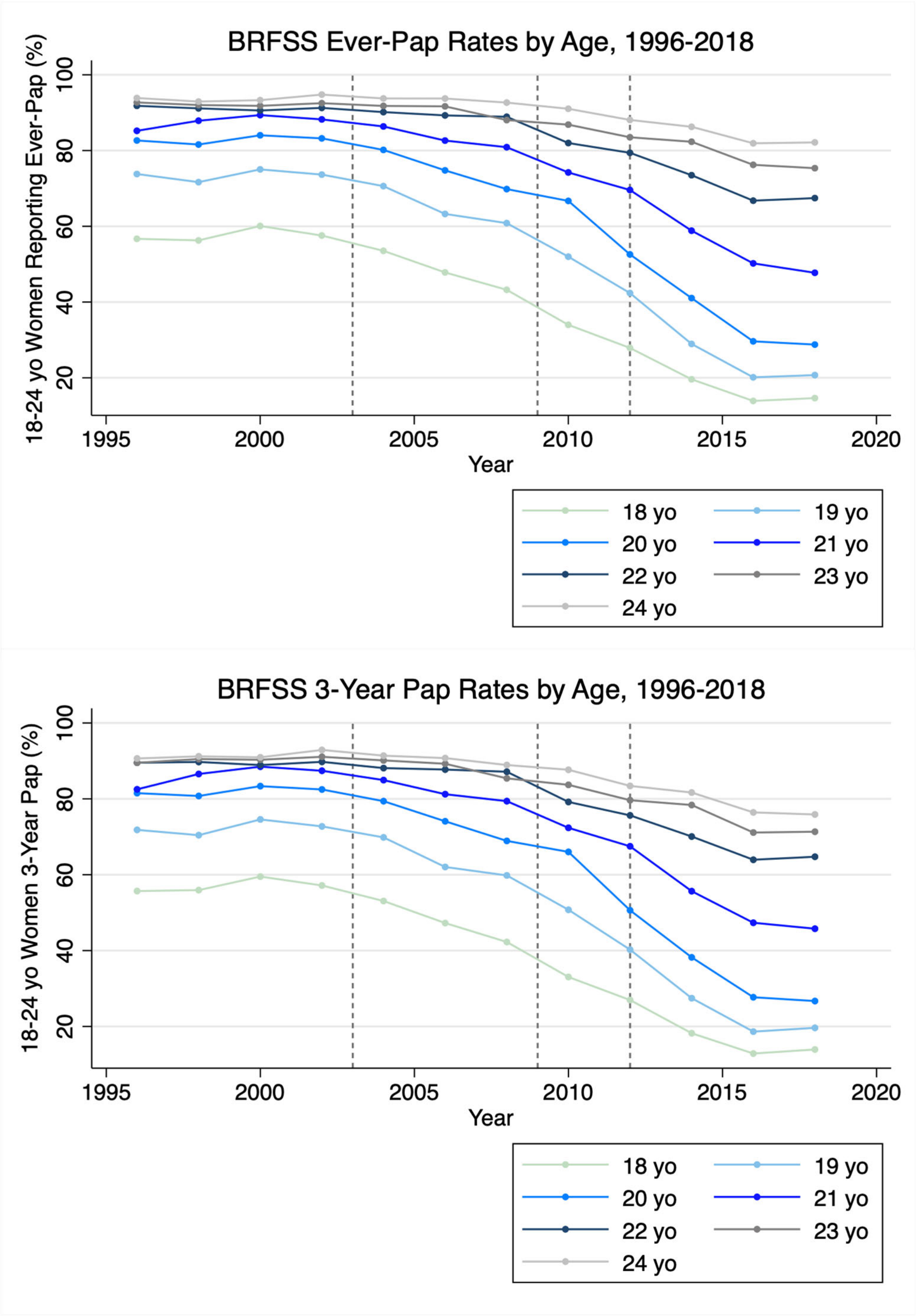

© 2023 Bromley-Dulfano RA et al. *JAMA Health Forum.*

eFigure 3. Cervical Cancer Screening Trends from 1996-2018 by Race and Ethnicity, Ever-Pap and 3-Year Pap Tests. The figure shows screening rates from BRFSS data representing the percentage of respondents who reported ever having received a Pap Test (Ever-Pap, top panel) or receiving a Pap test in the past three years (3-Year Pap, bottom panel) by race and ethnicity. Dashed vertical lines represent years of major ACOG guideline changes.

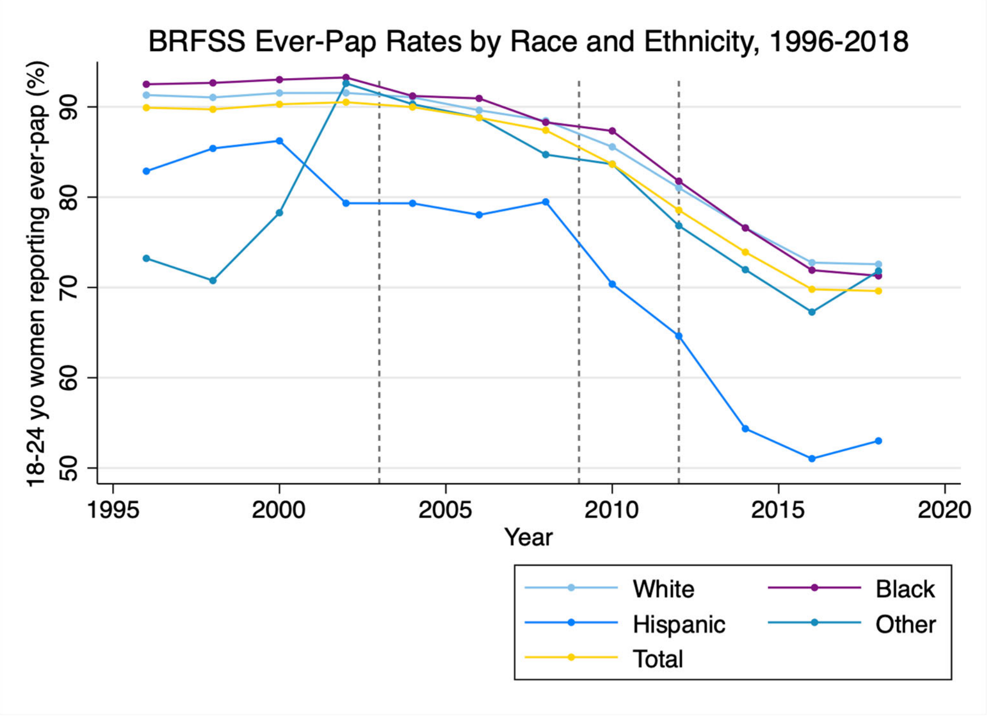

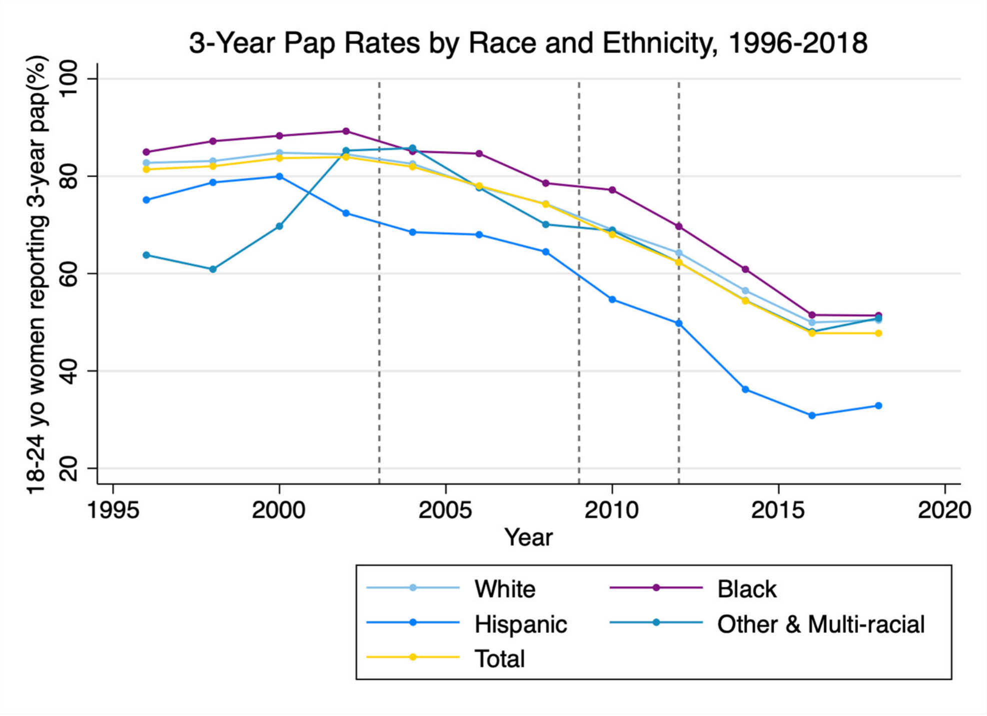

eFigure 4. BRFSS Screening Data Correlation with Recommended Screening Variable, Ever-Pap and 3-Year Pap Tests. The figure shows the correlation between the recommended number of screenings and the percent of individuals reporting having ever had a Pap Test (Ever-Pap) and or a Pap test in the previous 3 years (3-Year Pap) screening rates from BRFSS data.

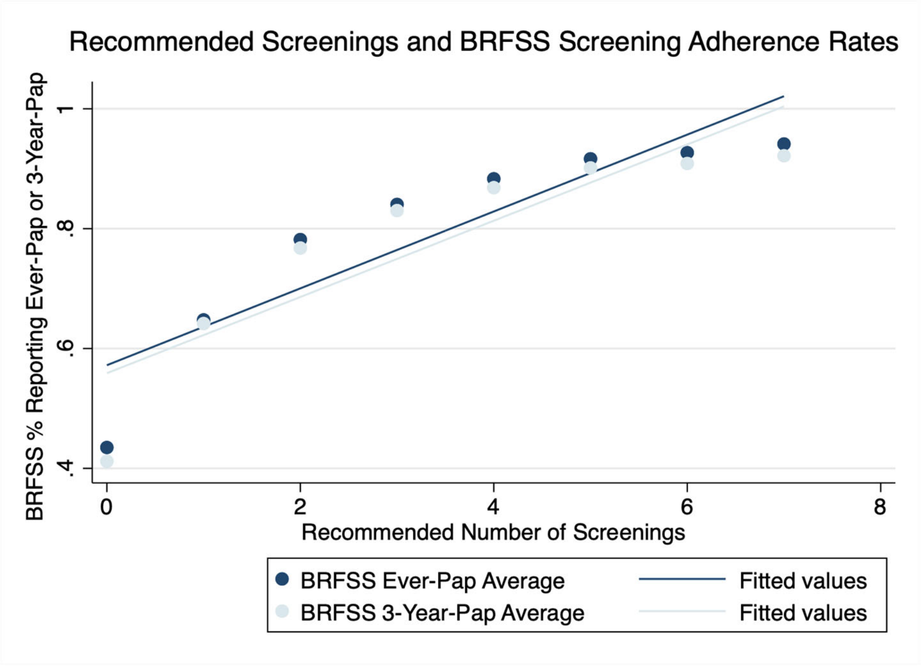

eFigure 5. BRFSS Screening Data Correlations with Recommended Screening Variable by Race and Ethnicity, Ever-Pap and 3-Year Pap. The figure shows the relationship between the recommended number of screenings and BRFSS Ever-Pap (top panel) and 3-Year Pap (bottom panel) screening rates stratified by race and ethnicity.

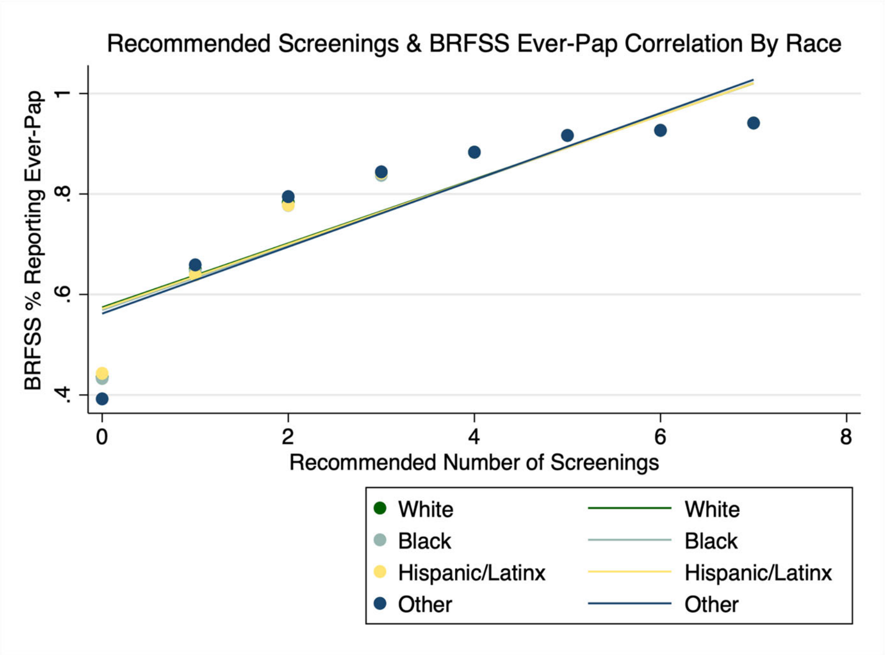

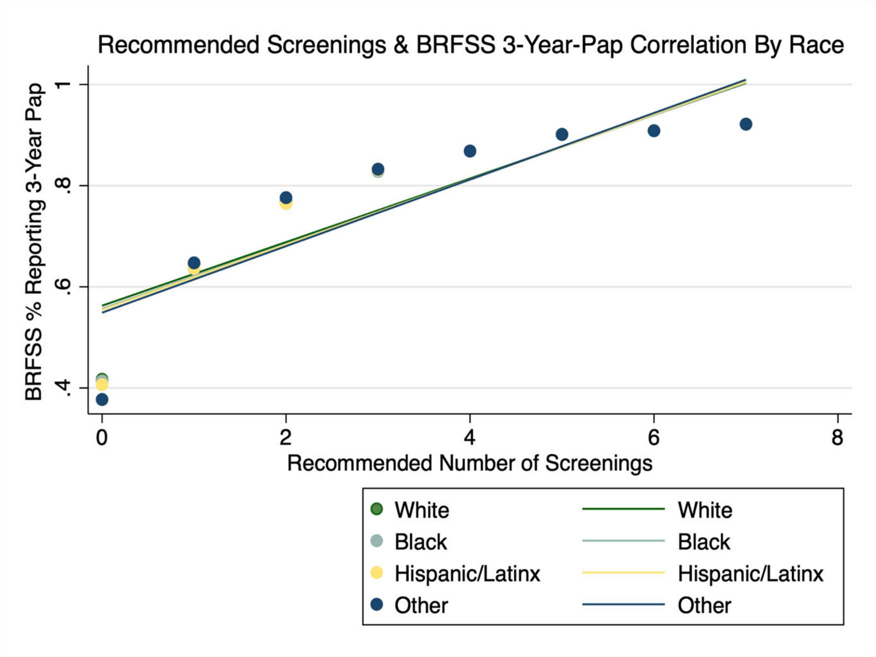

eFigure 6. Difference-in-Differences with Multiple Treatment Timings Event Study Graph. Event study graph shown for the binary treatment model reported in eAppendix 3. Controls are the same as those included as in the main model. Dark circles represent point estimates and the surrounding bars represent 95% CIs.

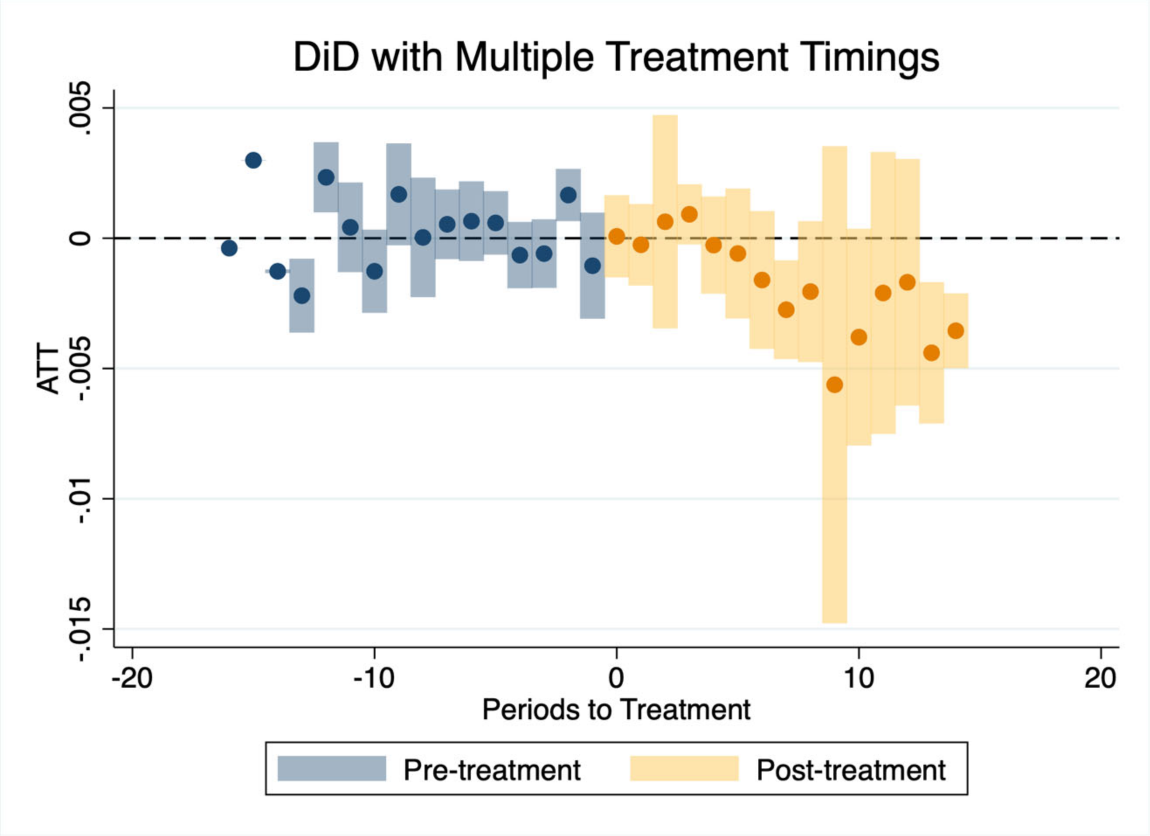

eReferences

1. Callaway B, Sant'Anna PHC. Difference-in-Differences with multiple time periods. *J Econom*. 2021;225(2):200-230. doi:10.1016/j.jeconom.2020.12.001

2. Sun L, Abraham S. Estimating dynamic treatment effects in event studies with heterogeneous treatment effects. *J Econom*. 2021;225(2):175-199. doi:10.1016/j.jeconom.2020.09.006

3. Hirth J. Disparities in HPV vaccination rates and HPV prevalence in the United States: a review of the literature. *Hum Vaccines Immunother*. 2019;15(1):146-155. doi:10.1080/21645515.2018.1512453
