# Supplementary material for: Association Between Cervical Cancer Screening Guidelines and Preterm Delivery Among Females Aged 18 to 24 Years
Source: JAMA Health Forum. 2023 Jul 21;4(7):e231974. doi: 10.1001/jamahealthforum.2023.1974 (PMC10362467; doi:10.1001/jamahealthforum.2023.1974)
Supplement: Supplement 2. — Data Sharing Statement [file jamahealthforum-e231974-s002.pdf]

Data Sharing Statement

Bromley-Dulfano. Association Between Cervical Cancer Screening Guidelines and Preterm Delivery Among Females Aged 18 to 24 Years. *JAMA Health Forum*. Published July 21, 2023. doi:10.1001/jamahealthforum.2023.1974

Data
Data available: Yes
Data types: Data (not involving human participants)
How to access data: We would be happy to share our data set at any point in time. Please email the Corresponding Author to request the data.
When available: With publication

Supporting Documents
Document types: Statistical/analytic code
How to access documents: Please email the Corresponding Author with any requests.
When available: With publication

Additional Information
Who can access the data: Anyone requesting the data
Types of analyses: For any purpose
Mechanisms of data availability: Data are all from publicly available sources and have been aggregated for this project, we would be happy to share our compiled dataset without restriction.
